# What Makes a Customer Brand Citizen in Restaurant Industry

**DOI:** 10.3389/fpsyg.2022.676372

**Published:** 2022-03-22

**Authors:** Hua Han, Yi-Chun Yang, Tingyue Kuang, Hemin Song

**Affiliations:** ^1^Sports Business School, Beijing Sport University, Beijing, China; ^2^Division of Business and Management, Beijing Normal University-Hong Kong Baptist University United International College, Zhuhai, China; ^3^Faculty of Business, City University of Macau, Macao, Macao SAR, China

**Keywords:** brand uniqueness, brand credibility, brand intimacy, brand love, brand citizenship behavior

## Abstract

Due to the crucial role of customers’ brand citizenship behaviors on brand strength, this study explored the relationship between brand uniqueness, brand credibility, brand intimacy, brand love, and brand citizenship behavior in Taiwan’s restaurant context. The participants are the customers of Wang Steak, a famous restaurant chain in Taiwan. A total of 358 valid responses were gathered from a questionnaire survey, with a response rate of 71.6%. We used structural equation modeling to analyze the data. Brand uniqueness, brand credibility, and brand intimacy all have a positive relationship with brand love. Moreover, brand love was positively associated with brand citizenship behavior. The findings provide greater insights into the relationship of perceived brand uniqueness, brand credibility, brand intimacy, and brand love with customers’ brand citizenship behaviors.

## Introduction

Brand management is important for the restaurant industry because brand management enables restaurant brands to maintain brand image perception among customers (Naehyun et al., 2012). With regard to brand area, it is also noteworthy that research on brand citizenship behavior has created its own area of brand study (Nyadzayo et al., 2015). Brand citizenship behavior comes from the nature of organizational citizenship behavior; it is defined as a voluntary behavior that benefits a brand (Morhart et al., 2009). That is, customers are willing to volunteer for the development of the firm’s brand. Customers play the role of a second employee because their behaviors are as much of a help to business development as employees (Ahn et al., 2016). Customers tend to perform cooperative behaviors and extra roles to support a brand (Ahn et al., 2016). Those with high brand citizenship behavior are more likely to share positive brand experiences with others (Yi et al., 2013), which enhances brand performance and brand strength (Nyadzayo et al., 2015).

In addition, brand love is a relatively well-known topic of research (Albert et al., 2008; Batra et al., 2012; Sajtos et al., 2021). Ahuvia et al. (2009, pp. 354–355) noted that “research on loved objects provides a spotlight for illuminating some of the most psychically significant consumption experiences (Renée et al., 2021). Brand love is beneficial for managing consumer-brand relationships (Bıçakcıoğlu et al., 2018), and it can create positive outcomes, including brand loyalty, positive word of mouth, and willingness to pay (Batra et al., 2012). Understanding the factors influencing brand love is extremely important as “a deeper understanding of how consumers feel, think, and act could provide valuable guidance to address” the challenges of brand management (Keller, 2003, p. 595).

Due to the important role of brand love (Bairrada et al., 2018), diversified theories have been used to understand the concept of brand love (Bigne et al., 2020). First, the brand love stream of research has seen frequent usage of social exchange theory. Social exchange theory describes the crucial exchanges between two parties or two partners that participate to produce a quality relationship (Wang et al., 2019). This theory can be applied to understand what it takes for a strong and satisfied consumer-brand relationship, which also exhibits the importance of this theory in consumer-brand relation outcomes such as brand love (Garg et al., 2016). Second, social identity theory is another extensively used theory in brand love literature. Social identity theory is useful in understanding the interaction of people in a social network (Tajfel et al., 1979), as they go beyond their personal identity to develop a social identity. This theory elucidates that a person’s positive self-esteem is enhanced through the person’s personal identity and/or social identity (Edwards, 2005), which at the end add up to the person’s self-concept, which is the sum total of the individual’s thoughts and feelings having reference to the individual as an object (Gumparthi and Patra, 2020). Third, attachment theory in this stream of research was used to understand the alignment of love-like feelings for brands to understand customers’ emotional attachment toward brands (Gumparthi and Patra, 2020). Despite the growing studies that used the related theories to understand brand love, it has not drawn much attention to use the theory of stimulus-organism-response (SOR) to explore factors influencing brand love.

To fill the gap, this research applied the concept of SOR to test the stimuli of brand uniqueness, brand credibility, and brand intimacy to enhance brand citizenship behavior mediated by brand love. SOR proposes that the environment is a stimulus (S), which consists of a set of signs that cause an individual’s internal evaluation (O) and then produces a response (R). The SOR model suggests that consumers’ emotions become an important part in responding to the exposing environmental stimulus (Deng and Yang, 2021). Based on this theory, we used SOR to propose that brand uniqueness, brand credibility, and brand intimacy are stimuli (S); it will cause an internal evaluation of someone (O) (brand love) and then produce a response (R) (brand citizenship behavior). This study developed a framework and proposed that brand uniqueness, brand credibility, and brand intimacy play important roles in determining brand love, which enhances brand citizenship behavior. We proposed four hypotheses, and data were collected from customers in Taiwanese restaurants. This study used a sample of Taiwanese studies to confirm its research structure through an empirical approach. This study takes Taiwan’s restaurant industry as the research object due to three main reasons. First, the service industry has been the driving force behind Taiwan’s economic growth in recent years, and the restaurant industry forms the growing proportion of the service industry. Second, the restaurant industry is a highly competitive industry, and the customers’ spontaneous altruistic spirit and citizenship behavior are beneficial to a restaurant’s competitive advantage. Third, unlike other countries, Taiwan’s restaurant industry is noted for asking employees to perform excellent service to customers, because the industry competition is fierce, and how to satisfy customers through the service process is an important part of value transfer.

This study advances the knowledge of brand through the following research contributions. First, the research extends our understanding of the concept of brand love through examining the roles of brand uniqueness, brand credibility, and brand intimacy on brand love. Second, the research analyzes the effects of brand love on brand citizenship behavior, which also adds to the knowledge about customers’ brand citizenship behavior, a concept that needs further attention. Third, the study examines the possible relationship between brand uniqueness, brand credibility, brand intimacy, brand love, and brand citizenship behavior in Taiwan’s restaurant context; we choose Wang Steak as the specific restaurant chain, because Wang Steak’s culture aims to generate customers’ brand love and brand altruistic behavior to enhance brand equity, which is suitable for the objectives of this study. The result can give us a deeper understanding of brand citizenship behavior in the restaurant context.

## Literature Review and Hypothesis

### Brand Uniqueness

Uniqueness means customers feel the brand is quite distinct from other brands (Aaker, 1996). Brand uniqueness can be judged from featured advertising claims or personal experience with a brand (Kalra and Goodstein, 1998). When a brand creates a unique image in the customer’s mind, it can generate a price premium in the market (Aaker, 1996). That is, brand uniqueness offers specific information by differentiating the brand from its competitors. The unique brand image provides customers a heuristic to choose among alternatives (Kalra and Goodstein, 1998).

### Brand Credibility

Brand credibility refers to the believability of the position information attached to a brand, based on customers’ perceptions of whether the brand can fulfill the promised commitment (Erdem and Swait, 2004). Brand credibility comprises two main components: trustworthiness and expertise (Sweeney and Swait, 2008). Trustworthiness means the extent to which the firms are willing to perform what they have promised. Expertise refers to the firms’ ability to deliver what they have promised. That is, brand credibility means whether or not the brand has the ability and the willingness to deliver what has been promised to customers (Erdem and Swait, 2004).

### Brand Intimacy

Brand intimacy is the degree to which customers perceive the brand cares about them and is willing to understand and serve their needs (Yim et al., 2008). A brand can truly capture the customers’ needs and preferences based on communication with the customers (Yim et al., 2008). This requires intense communication activities so that a brand can understand the customers’ current and potential needs (Kohli and Jaworski, 1990). By emphasizing the customers’ interests first, a brand can indicate to customers that it is committed to enhancing the well-being of the brand and holding an intimate relationship with its customers.

### Brand Love

Brand love refers to the degree of a person’s emotional attachment to a particular brand (Carroll and Ahuvia, 2006). Batra et al. (2012) further proposed that brand love is a type of relationship with a brand that comprises cognitive, affective, and behavioral factors. Those with brand love have a passion toward the brand (Bairrada et al., 2019); they generate positive emotional connections and entail overall positive evaluations with the brand, and they desire for the brand to become a part of their lives (Batra et al., 2012). That is, consumers regard the loved brand as part of themselves, which plays a crucial role in their world (Ahuvia et al., 2009).

### Brand Citizenship Behavior

The notion of customer citizenship behavior came from the theory of organizational citizenship behavior (OCB) (Nyadzayo et al., 2011). Customer citizenship behavior refers to voluntary and discretionary behaviors that are not required for the successful production and delivery of service, but such behavior can help the organizational performance (Groth, 2005). Extending the concept of OCB to customers will allow researchers and managers to extend existing management theory to better understand the role customers play when interacting with service organizations (Groth, 2005). Customers’ active participation in the co-production of the service to a large extent determines the success of the service outcome (Gruen, 1995).

Brand citizenship behavior is an employee’s brand altruistic behavior to enhance brand equity. It enables employees to automatically display brand altruistic behavior, such as good service behavior, which can improve the brand image (Sun et al., 2007). Although this concept refers to employee behaviors toward a brand, it can also be extended to customer behaviors because customers are more likely to support a brand displaying altruistic behaviors (Ahn et al., 2016). A prior study proposed that brand citizenship behavior can be divided into three constructs: (1) brand enthusiasm, (2) brand endorsement, and (3) helping behavior (Xie et al., 2014). Specifically, brand enthusiasm refers to customers’ engagement in additional brand activities, such as sharing customers’ views that strengthen branding options and participating in brand affairs. Brand endorsement is related to advocating a brand to relatives, friends, and other customers, which delivers positive word of mouth and is very crucial in competitive markets (Tuškej et al., 2013). Helping behavior is associated with customers’ positive actions toward a brand, which represents customers demonstrating friendliness toward a brand (Nyadzayo et al., 2015).

### The Relationship Between Brand Uniqueness and Brand Love

Brand uniqueness refers to “the degree to which customers feel the brand is different from competing brands” (Netemeyer et al., 2004, p. 211). Research has supported that perceived uniqueness generates customer utility, which contributes to the consumers’ preferences (Carpenter et al., 1994). The foundation of brand love lies in the ways in which the brand is different from other competing brands, which makes brand uniqueness a possible variable influencing brand love (Cristela et al., 2018). Hence, based on the above arguments, this study proposed the following hypothesis:

**Hypothesis 1:** Brand uniqueness has a positive effect on brand love.

### The Relationship Between Brand Credibility and Brand Love

Brand credibility refers to the consumers’ judgments about the believability of the information embedded by a brand. It indicates consumers’ perceptions of whether or not the brand has the ability and the willingness to deliver what has been promised (Erdem and Swait, 2004). Baek et al. (2010, p. 663) stated that brand credibility “provides unbeatable benefits to both consumers and companies.” Such a description is thus crucial for the development of brand love. Owing to imperfect and asymmetrical information, consumers merely depend on brand signals to make inferences about the qualities of a brand (Wernerfelt, 1988). Accordingly, brand credibility can be viewed as a signal to make consumers develop confidence in the brand’s attributes, hence generating brand love (Cristela et al., 2018). Therefore, according to the above arguments, this research suggested the following hypothesis:

**Hypothesis 2:** Brand credibility has a positive effect on brand love.

### The Relationship Between Brand Intimacy and Brand Love

Brand intimacy means the customers’ perception of the degree of concern that the brand has for them and its commitment to understanding the customers’ needs (Yim et al., 2008). It requires communication to truly capture the customers’ needs and preferences (Akçura and Srinivasan, 2005; Yim et al., 2008). Building intimacy relies on continuously working to bring “warmth” to a relationship (Sternberg, 1986), and it requires gaining knowledge “to truly understand its customers’ needs and preferences” (Akçura and Srinivasan, 2005, p. 1007). Customer intimacy arising from communication facilitates understanding of the needs and preferences of the customers, enabling firms to provide products or services that closely meet the customers’ needs and can generate customers’ brand love (Cristela et al., 2018). Consequently, brand intimacy appears to be a crucial determinant to trigger a brand love relationship. Accordingly, we predict the following:

**Hypothesis 3:** Brand intimacy has a positive effect on brand love.

### The Relationship Between Brand Love and Brand Citizenship Behavior

Loving a brand means a person has a passion toward the brand, generating positive emotional connections with the brand (Batra et al., 2012), which makes consumers strive to maintain the relationship with the brand. Previous research has supported that brand love contributes to brand loyalty, positive word of mouth, and willingness to pay a price premium (Batra et al., 2012). Customers who love a brand are expected to be more committed to the brand, spread “the good word” to others, and resist any negative information. By applying the nature of brand love, this study proposes that brand love is a positive brand involvement, and this may generate the display of brand altruistic behavior. Hence, we offer the following:

**Hypothesis 4:** Brand love has a positive effect on brand citizenship behavior.
**Hypothesis 4a:** Brand love has a positive effect on brand enthusiasm.
**Hypothesis 4b:** Brand love has a positive effect on brand endorsement.
**Hypothesis 4c:** Brand love has a positive effect on helping behavior.

## Methodology

### Participants

The participants of the study were customers. During the COVID-19 pandemic, because the situation in Taiwan is under control, most people are willing to eat out, and everyone wore a mask to avoid the impact of COVID-19. We used a questionnaire survey to gather data for analysis. The participants were the customers of Wang Steak, a famous restaurant chain in Taiwan. Wang Steak provides attentive service, comfortable dining environment, and warm lighting and uses green plants for decor to give customers a satisfying dining experience.

### Procedure

Prior to the formal survey, the questionnaires were sent to the customers of Wang Steak, and forty people were chosen for pretest to validate the ambiguities and meanings of the questionnaire items. After that, this research can confirm that the scales have previously been demonstrated to be valid and reliable for use with this population. The research then used convenience sampling, and 500 questionnaires were sent to the customers of Wang Steak. The surveys were administered while the customers were waiting to complete their payments after the meal. We offered all participants a free beverage along with a $5 gift certificate that can be used in Wang Steak. Researchers gave questionnaires together with cover letters to all the participants. The cover letter guaranteed that all responses would be kept confidential. In addition, to ensure anonymity, all completed surveys were anonymous and returned to the researchers directly after they finish the survey.

A total of 358 effective responses were gathered (a response rate of 71.6%). With regard to the sample, 62.4% were female participants, 59.8% had bachelor’s degrees, the mean age was 27 years (SD = 1.21), and the mean salary was NT 35,000 (SD = 12,909).

### Measures

A five-point Likert-type scale anchored at 1 = “strongly disagree” and 5 = “strongly agree” was applied to measure the respondents’ perceptions.

#### Brand Uniqueness

The scale of brand uniqueness was formed by using the four-item scale proposed by Richard et al. (2004). Sample items included “(Brand name) is distinct from other brands of (product).” The coefficient alpha for this scale was 0.91.

#### Brand Credibility

Brand credibility was measured by using the 6-item scale proposed by Erdem and Swait (1998). Sample items included “This brand delivers what it promises.” The coefficient alpha for this scale was 0.93.

#### Brand Intimacy

The scale of brand intimacy was formed by using the three-item scale developed by Yim et al. (2008). Sample items included “This brand cares about customers’ preference.” The coefficient alpha for this scale was 0.92.

#### Brand Love

This study measured brand love using the instrument proposed by Bagozzi et al. (2016). Sample items included “I love this brand.” The coefficient alpha for the brand love scale in this study was 0.92.

#### Brand Citizenship Behavior

This scale was adopted from Burmann and Zeplin (2005) and comprised three dimensions: brand delivery (three items), brand enthusiasm (three items), and brand endorsement (three items). The alpha coefficients of brand delivery, brand enthusiasm, and brand endorsement were 0.92, 0.93, and 0.91, respectively.

### Analytical Strategy

This research examined the measurement model *via* LISREL VIII (Jöreskog and Sörbom, 1996). CFA of the measurement model was conducted before investigating the existing relationship between constructs in the hypothesized model. The overall measurement model with all constructs was estimated through CFA. The result shows that the model-fit indices of the CFA resulted in a good fit: chi-square value/d.f. = 2.24; RMSEA = 0.069; CFI = 0.91; IFI = 0.92; NFI = 0.91.

## Results

### Reliability and Validity

The reliability of all measures used in the survey was examined using Cronbach’s alpha coefficient; the results indicated that all the scales’ reliabilities were above the acceptable level of 0.7, indicating satisfactory reliability (Nunnally, 1978). Regarding construct validity, the average extracted variances (AVE) for each construct were all above 0.50 (ranging from 0.58 to 0.6) (Table 1), suggesting suitable convergent validity (Fornell and Larcker, 1981). This research then tested discriminant validity by using the correlations between the measures. As observed in Table 1, the main constructs were more strongly related to their own measures than with the others, and the square root AVE was greater than the off-diagonal elements in the corresponding rows and columns, thus confirming the discriminant validity (Fornell and Larcker, 1981). These results suggested that the measures used in this study possessed adequate reliability and validity.

**TABLE 1:** Descriptive statistics and correlations among indicator variables.

Variables	M	SD	AVE	(1)	(2)	(3)	(4)	(5)	(6)	(7)
Brand uniqueness (1)	4.18	0.89	0.64	**0.802**						
Brand credibility (2)	4.32	0.74	0.62	0.424**	**0.774**					
Brand intimacy (3)	4.23	0.71	0.59	0.356**	0.434**	**0.762**				
Brand love (4)	4.24	0.82	0.63	0.312**	0.324**	0.404**	**0.784**			
Brand enthusiasm (5)	4.21	0.72	0.59	0.328**	0.402**	0.487**	0.457**	**0.752**		
Brand endorsement (6)	4.19	0.64	0.61	0.414**	0.406**	0.398**	0.405**	0.438**	**0.712**	
Helping behavior (7)	4.17	0.83	0.58	0.436**	0.312**	0.417**	0.384**	0.412**	0.415**	**0.741**

*n = 358. Figures along the diagonal (in bold) are the values for the square root of the AVE.*

**p < 0.05.

**FIGURE 1:**
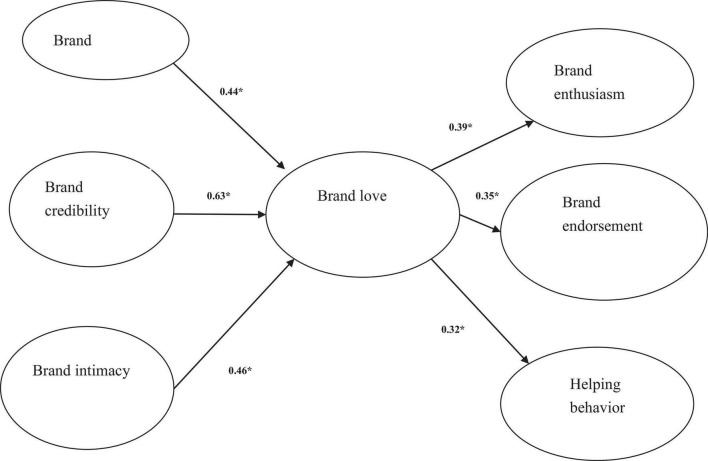
Conceptual model. Standardized path coefficients are reported; *p < 0.05.

After confirming the reliability and validity of the variables, we obtained an acceptable fit (chi-square value/d.f. = 2.428; RMSEA = 0.072; CFI = 0.93; IFI = 0.93; NFI = 0.92). The results of the overall fit measures suggested that the model (Figure 1) fits the data well. Having established an excellent fit between the structural model and the data, this research then tested the hypotheses on the basis of its parameter estimates.

### Hypothesis Testing

The 95% CI is used for interpreting the result of this study. H1 to H3 predicted that brand uniqueness, brand credibility, and brand intimacy can positively influence brand love. The results showed that brand love was significantly related to brand uniqueness (coefficient = 0.44, *p* < 0.01), brand credibility (coefficient = 0.63, *p* < 0.01), and brand intimacy (coefficient = 0.46, *p* < 0.01); thus, H1 to H3 were supported (Table 2).

**TABLE 2:** Results of hypotheses and model statistics.

	Path coefficient	*t*-Value	Results
Brand uniqueness → Brand love (H1)	0.44	3.99*	Supported
Brand credibility → Brand love (H2)	0.63	4.04*	Supported
Brand intimacy → Brand love (H3)	0.46	2.14*	Supported
Brand love → Brand enthusiasm (H4a)	0.39	3.95*	Supported
Brand love → Brand endorsement (H4b)	0.35	2.18*	Supported
Brand love → Helping behavior (H4c)	0.32	2.23*	Supported

*p < 0.05.

H4 posited that brand love can positively influence brand citizenship behaviors. The results supported that brand love significantly influenced brand enthusiasm (coefficient = 0.39, *p* < 0.01), brand endorsement (coefficient = 0.35, *p* < 0.01), and helping behavior (coefficient = 0.32, *p* < 0.01), thus supporting H4 (Table 2).

## Discussion

Given the importance of brand citizenship behavior to restaurant management, understanding ways in which this consumer behavior can be increased is a crucial part of a successful marketing strategy. In sum, this study used the concept of SOR to test the stimuli of brand uniqueness, brand credibility, and brand intimacy in generating brand love and brand citizenship behavior in restaurant’s consumers. The obtained results clearly support the predictions of the research model and have crucial academic and practical implications. The findings of this investigation uncovered the crucial effects of brand uniqueness, brand credibility, and brand intimacy on brand love, which demonstrated that both functional and emotional or symbolic elements are important to the development of brand love.

Specifically, brand uniqueness is a possible factor influencing brand love, the results of which are consistent with the finding of prior research (Cristela et al., 2018). Cristela et al. (2018) found that the uniqueness of brand enables customers to generate brand love toward the brand, implying that brand uniqueness is a possible determinant influencing brand love. Regarding brand credibility, the results tend to be consistent with our predictions. Brand credibility can be regarded as a useful tool to enable consumers to develop confidence in the brand’s attributes; therefore, they can generate brand love (Cristela et al., 2018). Finally, brand intimacy relates positively to brand love, congruent with the findings of Cristela et al. (2018). Breivik and Thorbjørnsen (2008) suggested that brand intimacy is the customers’ perception about the brand concerning their needs; this study further indicated that this perception is likely to convey emotional and symbolic benefits to customers, leading to the development of a love relationship.

Moreover, brand citizenship behavior is the outcome of brand love in Taiwan’s restaurant setting. Brand citizenship behavior is considered a discretionary behavior, implying that employees tend to involve public affairs rather than individual ones for a certain brand (Yi et al., 2013). Those with brand love tend to perform brand enthusiasm, brand endorsement, and helping behavior. The proposed relationship between brand love and brand citizenship behavior offers a greater understanding of customers’ responses to various brands, which enables scholars to better understand brand citizenship behaviors (Nyadzayo et al., 2015).

This study furthers the research field by examining the influences of brand love on three types of brand citizenship behavior and ultimately contributes to the existing brand literature by investigating the distinct dimensions of brand citizenship behavior as outcomes of brand love in the restaurant context.

In addition to theoretical implications, this study provides managers with some insights on how to spur brand love and brand citizenship behavior. First, managers should focus on building a brand with unique traits, as this can deliver symbolic attributes to customers. Managers can do this in many different ways, such as price formulation, distribution, product features, and brand communication. Restaurant managers should consider improving the quality of their brands different from other competitors, bringing distinguished offerings to market, such as unique food and services that deliver new brand experiences. In addition, maybe firms can conduct food innovation to convey unique brand traits to customers. Second, restaurant managers should carefully fulfill their promises to customers so that they are able to deliver the believability of the information embedded by a brand in a systematic way, thus strengthening their brand’s credibility. For example, restaurants can guarantee the time of food delivery, and they really do very well in food delivery. Third, it is also important to develop intimate relationships with customers, and firms should put more effort into developing a deeper understanding of the customers’ needs and strive to fulfill those needs. Putting customers’ interests first is immensely helpful in creating brand intimacy. Restaurants can develop an intimate relationship with customers by establishing diversified channels of communication, such as the usage of virtual community to capture customers’ needs, and this may help keep intimate relationships.

Although the proposed conceptual model explains factors influencing brand citizenship behaviors, opportunities for further research remain. First, this study focuses on the effects of three brand traits on brand love and brand citizenship behavior. It is possible that there are other additional factors that might be potential determinants, such as the extent of brand innovation. In addition, future research can use an experimental design for exploring the causality of variables. Second, the generalizability of these research findings is limited as the research context is focused on Taiwan’s restaurant industry. The cross-sectional nature of this study may be a limitation. However, this does provide an excellent opportunity for researchers to compare the results with other cultural settings. Follow-up studies could be conducted with participants from other cultures, thereby increasing the validity and generalizability of our findings. Finally, the use of a limited industry category (i.e., restaurant industry) limits the generalizability of the findings to other service contexts. Because our sample was a convenience sample and participants were from restaurant’s customers in Taiwan, our findings might not generalize to all restaurant’s customers in other industries and other countries. Hence, future research might want to examine the research model’s applicability to other service contexts, such as the retailing industry.

## Data Availability Statement

The datasets presented in this article are not readily available because, we used questionnaire survey to collect primary data. Requests to access the datasets should be directed to Y-CY, frankie130811@gmail.com.

## Ethics Statement

The studies involving human participants were reviewed and approved by Division of Business and Management, Beijing Normal University-Hong Kong Baptist University United International College. The patients/participants provided their written informed consent to participate in this study.

## Author Contributions

All authors contributed to conception and design of the study, database collection, statistical analysis, draft of the manuscript, manuscript revision, and read and approved the submitted version.

## Conflict of Interest

The authors declare that the research was conducted in the absence of any commercial or financial relationships that could be construed as a potential conflict of interest.

## Publisher’s Note

All claims expressed in this article are solely those of the authors and do not necessarily represent those of their affiliated organizations, or those of the publisher, the editors and the reviewers. Any product that may be evaluated in this article, or claim that may be made by its manufacturer, is not guaranteed or endorsed by the publisher.
